# Dr. John Burns

**Published:** 1910-04-02

**Authors:** 


Dr. John Burns, one of the oldest medical practitioners in the country, died at Glasgow on Thursday night. Dr. Burns, who was in his 95th year, continued the practice of his profession until five years ago. He was a native of Perth, and in 1846 became a licentiate of the Glasgow College of Physicians and Surgeons. Five years later, in 1851, he was elected a Fellow of the Faculty, of which he was subsequently for a term vice-president. In early life, when medical attendant to a distinguished invalid, he visited the chief cities of Europe, Egypt, and the Holy Land. For sixty years he was one of the most successful and best-known medical practitioners in Glasgow and the West of Scotland. His portrait, painted on behalf of a large number of subscribers, was presented to the Glasgow Corporation, and now occupies a place in the People's Palace on Glasgow Green, in the immediate neighbourhood of the district in which he practised for so many years.

